# The Effect of Discharge Mode on the Distribution of Myocardial Pulsed Electric Field—A Simulation Study for Pulsed Field Ablation of Atrial Fibrillation

**DOI:** 10.3390/jcdd9040095

**Published:** 2022-03-24

**Authors:** Xingkai Ji, Hao Zhang, Lianru Zang, Shengjie Yan, Xiaomei Wu

**Affiliations:** 1 Centre for Biomedical Engineering, School of Information Science and Technology, Fudan University, Shanghai 200433, China; 20210720130@fudan.edu.cn (X.J.); 20210720044@fudan.edu.cn (H.Z.); 21110720131@m.fudan.edu.cn (L.Z.); 2 Academy for Engineering and Technology, Fudan University, Shanghai 200433, China; 3 Key Laboratory of Medical Imaging Computing and Computer-Assisted Intervention (MICCAI) of Shanghai, Fudan University, Shanghai 200433, China; 4 Shanghai Engineering Research Centre of Assistive Devices, Shanghai 200433, China; 5 Yiwu Research Institute, Fudan University, Chengbei Road, Yiwu City 322000, China

**Keywords:** pulsed electric field ablation, finite element simulation, discharge mode

## Abstract

Background: At present, the effects of discharge modes of multielectrode catheters on the distribution of pulsed electric fields have not been completely clarified. Therefore, the control of the distribution of the pulsed electric field by selecting the discharge mode remains one of the key technical problems to be solved. Methods: We constructed a model including myocardium, blood, and a flower catheter. Subsequently, by setting different positive and ground electrodes, we simulated the electric field distribution in the myocardium of four discharge modes (A, B, C, and D) before and after the catheter rotation and analyzed their mechanisms. Results: Modes B, C, and D formed a continuous circumferential ablation lesion without the rotation of the catheter, with depths of 1.6 mm, 2.7 mm, and 0.7 mm, respectively. After the catheter rotation, the four modes could form a continuous circumferential ablation lesion with widths of 10.8 mm, 10.6 mm, 11.8 mm, and 11.5 mm, respectively, and depths of 5.2 mm, 2.7 mm, 4.7 mm, and 4.0 mm, respectively. Conclusions: The discharge mode directly affects the electric field distribution in the myocardium. Our results can help improve PFA procedures and provide enlightenment for the design of the discharge mode with multielectrode catheters.

## 1. Introduction

Atrial fibrillation (AF) is a common arrhythmia in which the incidence rate increases with age [1,2]. To date, ablation has become one of the most effective methods for the treatment of AF, and circular pulmonary vein vestibular isolation is its basic operation [3]. The forms of ablation energy include radio frequency, freezing, and pulsed electric field. The first two energy forms for producing ablation are based on temperature, and it is easy to damage the adjacent tissues around the ablation target and to produce serious complications [4]. As a new nonthermal ablation method, pulsed electric field ablation (PFA) has the advantage of tissue selectivity and has attracted extensive attention [5,6,7].

PFA uses a series of high-intensity electrical pulses to produce a large number of water-soluble holes in the cell membrane, which changes the cell permeability; additionally, some substances can pass in and out of the cell through these holes. When the pulse intensity reaches a certain threshold, the holes in the cell membrane cannot be restored after ablation, and irreversible electroporation (IRE) [8,9] is formed. IRE leads to apoptosis and can be used for AF ablation. Studies have shown that when the electric field intensity reaches 400 V/cm, IRE will occur in the myocardium [5,10].

In AF ablation, ablation lesions of different sizes and forms, such as rings, lines, and points, may be needed, according to the actual situation. A number of studies have shown that the size and shape of ablation lesions that are caused by PFA are not only controlled by electrical parameters [11,12,13], such as the pulse amplitude, pulse width, pulse number, and pulse interval, but can also be affected by electrode structure [14]. Recently, many laboratories have conducted clinical trials of PFA. Stewart et al. [15] used a multielectrode loop catheter to perform intracardiac PFA in six pigs with a bidirectional pulse sequence. Two weeks after the operation, it was found that PFA caused uniform myocardial lesions. The activity of the cells around the target was retained, whereas the target cardiomyocytes were apoptotic. Yavin et al. [16] explored the PFA of the right atrium from the superior vena cava to the inferior vena cava in 12 pigs using the bidirectional circular catheter “Lasso PFA” and a bidirectional pulse sequence. After PFA, all 12 pigs formed acute block lines with no observable effects on the esophagus and phrenic nerve. Reddy et al. [17] conducted a clinical trial on 81 patients with paroxysmal atrial fibrillation by using a flower catheter and a monophasic/biphasic pulse sequence. The pulmonary vein isolation time of all the patients was less than 3 min, and the immediate pulmonary vein isolation rate reached 100%. Moreover, there were no other major adverse reactions (except for pericardial tamponade in one case).

These clinical trials of PFA provide the results of different ablation strategies, but the mechanisms involved need to be further elucidated, whereas modeling and simulation can solve this problem. Guo et al. [18] used a finite element model (COMSOL) to study the effects of the dielectric dispersion and anisotropic conductivity of tissues during electroporation pulses. Merola et al. [19] conducted a theoretical analysis of PFA using COMSOL and explored the influence of different applied voltages and different geometries of electrodes on the electric field distribution. Mercadal et al. [20] compared the efficiency of electroporation and the ability to trigger neural action potentials of different pulse protocols using a numerical analysis method (COMSOL).

For multielectrode ablation catheters such as flower catheters (Farawave^TM^, Farapulse, Natick, MA, USA) [21], different discharge modes can change the electric field distribution in the myocardium, thus resulting in the superposition or cancellation of the electric field and the subsequent phenomenon of the ablation effect of PFA in the target tissue region. Therefore, clarifying the relationship between the discharge mode and electric field distribution in the myocardium has important guiding significance for clinical PFA mode setting.

We conducted a simulation study to improve PFA procedures by optimizing the discharge mode when implementing PFA with multielectrode catheters. The relationship between the discharge mode and electric field distribution in the myocardium was discussed in terms of electric field superposition and cancellation.

## 2. Methods

### 2.1. Construction of the Finite Element Model of Myocardial PFA

**Figure 1:**
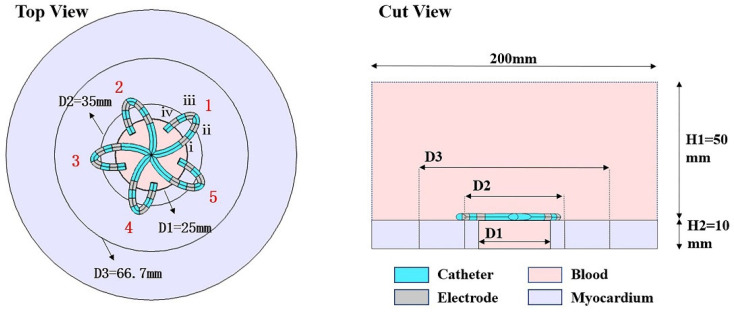
Flower catheter structure and myocardial and blood tissue model. Blood thickness H1 = 50 mm, myocardial thickness H2 = 10 mm, pulmonary vein diameter D1 = 25 mm. The electrode spacing was 2.5 mm, the electrode length was 2.5 mm, and the electrode diameter was 2.33 mm. The gray area in the model is the electrode, the light blue area is the catheter, the lavender area is the myocardium, and the light pink area is the blood. The red numbers of “1–5” in the top view are the serial numbers of the catheter spline, whereas D2 and D3 are the auxiliary lines. “i–iv” represent the serial numbers of the four electrodes on spline 1, and the other splines are also numbered “i–iv” in the same order.

Based on the flower catheter (referred to as the structure of Farapulse, and the dimensions and geometry are not exactly the same), we established a finite element model of myocardial PFA by using COMSOL Multiphysics 5.6 (COMSOL, Burlington, MA, USA), as shown in Figure 1. The flower catheter is composed of 5 splines, with 4 platinum electrodes on each spline. Due to the low requirement of PFA for electrode–tissue contact [22], the model was simplified in our study by horizontally attaching the catheter to the myocardium surrounding the pulmonary vein. The atrial wall thickness and pulmonary vein diameter are between 2–3 mm [23,24] and 15–25 mm [25], respectively. To fully reflect the distribution of the electric field and to make the simulation results more universal, we set the myocardial thickness to 10 mm and the pulmonary vein diameter to 25 mm. The blood covered the entire catheter and was set at a height of 50 mm to simulate the situation of filling the left atrium with blood (the diameter of the left atrium is 40–50 mm [26]). The whole-model diameter was set to 200 mm to avoid the influence of electrical insulation conditions at the model boundary on the myocardium. However, we only focused on the myocardium around the pulmonary vein orifice; thus, in the follow-up results, only the electric field distribution in the range of D1–D3 is shown. The finite element model performed mesh refinements until the calculation error was less than 1%, and the final model consisted of 5,346,467 domain elements, 135,292 boundary elements, and 4221 edge elements.

The distribution of the electric field and potential in myocardial tissue can be obtained by solving the current continuity Equation (1), where J is the current density ($A/m^{2}$), ϕ is the potential (V), σ is the conductivity of the material (S/m), and $\varepsilon =\varepsilon_{0}\varepsilon_{r}$ is the dielectric constant of the material (where $\varepsilon_{0}$ is the dielectric constant in vacuum, and $\varepsilon_{r}$ is the relative dielectric constant of the material).

(1) $$\{\begin{array}{c} \nabla \cdot J=0\\ J=\sigma E+\frac{\partial \varepsilon E}{\partial t}\\ E=-\nabla \phi \end{array}$$

**Figure 2:**
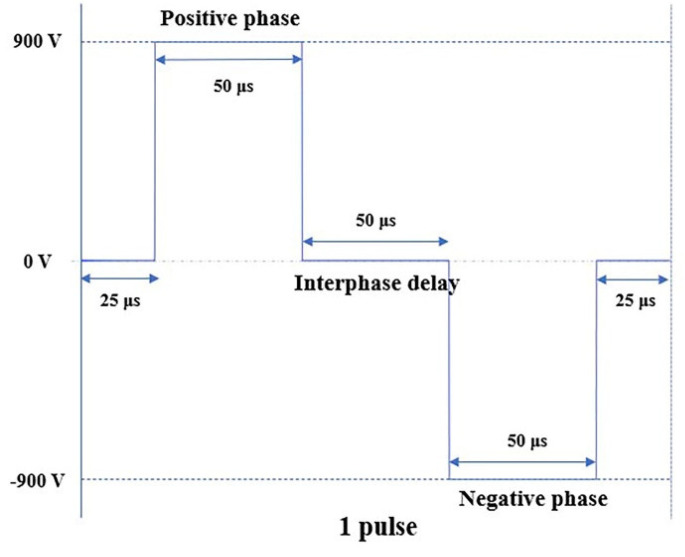
Biphasic pulse sequence.

To simplify the research process, we ignored the thermal effect of PFA [27] and set the conductivity of the myocardium and blood to static and isotropic. Based on these conditions, the number of pulses cannot affect the electric field distribution in the myocardium [28]. Therefore, only one biphasic pulse with an amplitude of 1800 Vp-p was applied to the electrodes in this manuscript. The positive phase, negative phase, and interphase delay of the biphasic pulse are 50 μs (as shown in Figure 2).

### 2.2. Simulation

Inspired by [29], we proposed three discharge modes (A, B, and C) and compared them with mode D from [30]. Due to the special structure of the flower catheter, a unified rotation strategy based on modes A, B, C, and D was developed to form a better continuous and transmural circumferential lesion. With the midpoint of the flower catheter as the center, the catheter was rotated counterclockwise twice (with rotations of 24° each time). The criterion to judge a discharge mode was whether it could form a uniform, continuous, and transmural circumferential lesion around the pulmonary vein orifice. In addition, unless otherwise specified, “the electric field distribution” refers to “the electric field distribution in the myocardium”.

**Figure 3:**
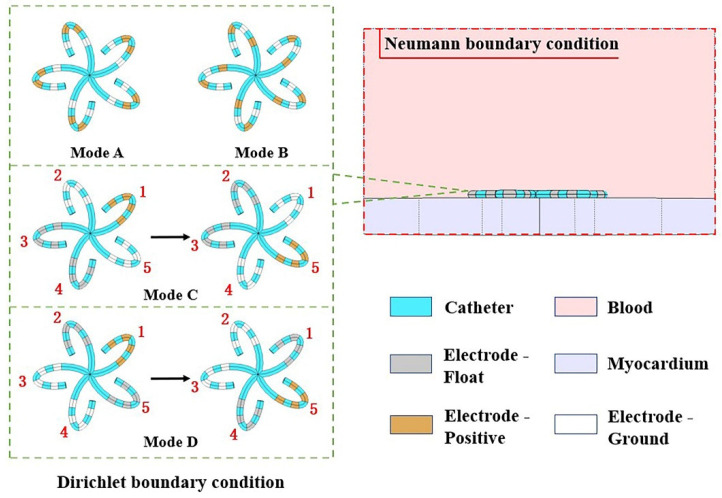
The boundary conditions of the model and the four discharge modes of the flower catheter.

Modes A and B are both simultaneous discharge modes, in which the adjacent electrodes on each spline of the flower catheter are simultaneously applied pulses in the way of “one positive and one ground”. Modes C and D are both sequential discharge modes, in which pulses are applied to the spline unit of the flower catheter in sequence. The electrode excitation corresponding to the above four discharge modes is the Dirichlet boundary condition of the model in this manuscript. In addition, the outer boundary of the whole model is described by the Neumann boundary condition as Equation (2), where n is the outer normal direction of the boundary. The specific settings of the positive and ground electrodes of the four discharge modes and the boundary conditions are shown in Figure 3.

(2) $$\frac{\partial \phi }{\partial n}=0$$

In Figure 3, “yellow” represents the positive electrodes, “white” represents the ground, and “gray” represents the float. In mode C, we first selected the four electrodes on spline 1 as the positive electrodes and then ground the eight electrodes on adjacent splines 2 and 5. After the electrical pulse was applied, the discharge spline was changed to a counterclockwise direction so that the positive electrodes were successively shifted to the “1–5” splines. Each time, two adjacent splines were selected to be grounded. Mode D is similar to mode C, except for the fact that the selection of the grounded spline was different. When spline 1 was selected as the positive electrodes, spline 3 and spline 4 were grounded. When spline 2 was selected as the positive electrodes, spline 4 and spline 5 were grounded. This process continued until all five splines were selected as the positive electrodes.

The material properties of the biological tissues and catheters at 5 kHz [31] are shown in Table 1.

## 3. Results

### 3.1. Electric Field Distribution without Catheter Rotation

**Figure 4:**
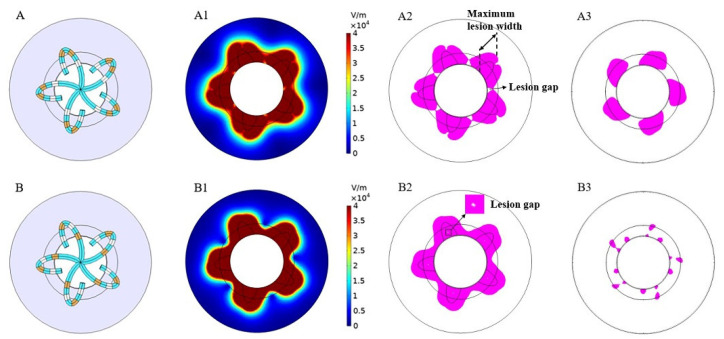
The electric field distribution and effective lesion area of modes (**A**,**B**) (without rotation). (**A1**,**B1**) is the electric field intensity distribution, and (**A2**,**B2**) and (**A3**,**B3**) are sectional views of the effective lesion area (magenta) on the myocardial surface and 3 mm below the myocardial surface, respectively.

**Figure 5:**
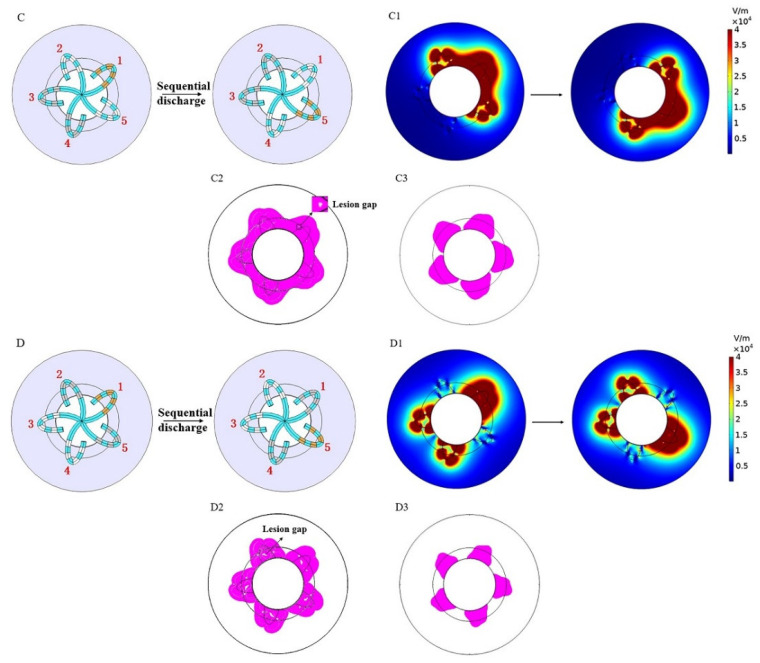
The electric field distribution and effective lesion area of modes (**C**,**D**) (without rotation). (**C1**,**D1**) is the electric field intensity distribution, and (**C2**,**D2**) and (**C3**,**D3**) are sectional views of the effective lesion area (magenta) on the myocardial surface and 3 mm below the myocardial surface, respectively.

In this manuscript, the area where the electric field strength E ≥ 400 V/cm was referred to as the effective lesion area (ELA), and since the actual atrial wall thickness is in the range of 2–3 mm, to better reflect the ablation effect and wall penetration, we showed the ablation situation both on the myocardial surface and 3 mm below under different discharge modes. Figure 4 and Figure 5show the simulation results of mode A to mode D, respectively (without rotation). The results include electric field distribution and ELA. ELA is represented by magenta and the rest is represented by white.

It can be seen in Figure 4 that, compared with mode B, mode A had a deeper lesion and a larger ELA 3 mm below the myocardial surface; however, the lesion on the myocardial surface was discontinuous. It should be noted that there were several lesion gaps (indicated by small white spots of E < 400 V/cm, as shown in Figure 4B2) on the myocardial surface in mode B, which is a reflection of uneven PFA and may have some adverse effects on complete isolation.

Figure 5 shows the simulation results of modes C and D. Compared with modes A and B, modes C and D formed a continuous circumferential ELA on the myocardial surface and also created a large area of ELA 3 mm below the myocardial surface. Compared with mode D, the ELA caused by mode C was larger both on the surface and 3 mm below the myocardium, and the lesion gap of mode C was also significantly smaller than that of mode D (as shown in Figure 5C2,D2).

Table 2 shows the dimensions of the ELA of the four discharge modes without catheter rotation. Modes A and C caused the greatest myocardial lesion depth (6.1 mm) and width (11.8 mm), respectively. Among the four discharge modes, modes B, C, and D could achieve a certain lesion depth while maintaining the continuity of myocardial lesion, and their corresponding maximum continuous circumferential lesion depths were 1.6 mm, 2.7 mm, and 0.7 mm, respectively.

### 3.2. Electric Field Distribution with Catheter Rotation

**Figure 6:**
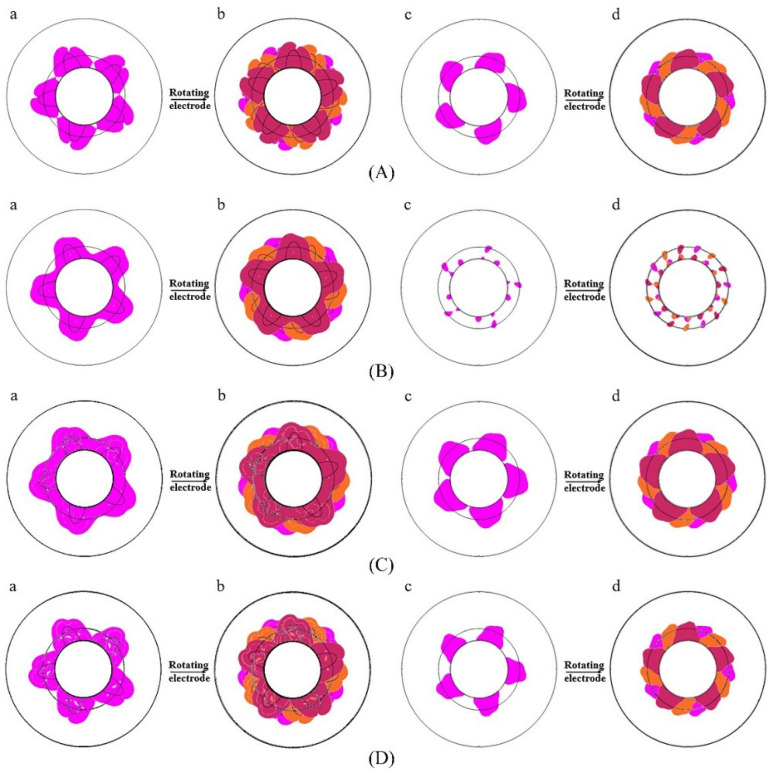
The effective lesion area after rotation. (**A**–**D**) represent the discharge modes A–D, respectively. (**a**) and (**c**) are the effective lesion areas caused by a single ablation, (**a**) and (**b**) are on the surface of the myocardium, and (**c**) and (**d**) are 3 mm below the myocardium. (**b**) and (**d**) are the total effective lesion areas caused by the corresponding two rotations. Magenta represents the effective lesion area before rotation, and orange and dark red represent the effective lesion area after rotations of 24° and 48°, respectively.

Figure 6 shows the simulation results of the four discharge modes after two rotations. All four discharge modes (except mode B) formed a continuous ELA 3 mm below the myocardium. Table 3 lists the maximum continuous circumferential lesion depth after two rotations for the four discharge modes. Since the width and depth of the maximum myocardial lesion caused by catheter rotation did not change, they were not listed in Table 3. The data in Table 3 show that mode A has the largest continuous circumferential lesion depth after two rotations (5.2 mm).

## 4. Discussion

### 4.1. Influencing Factors of Electric Field Distribution in the Myocardium

**Figure 7:**
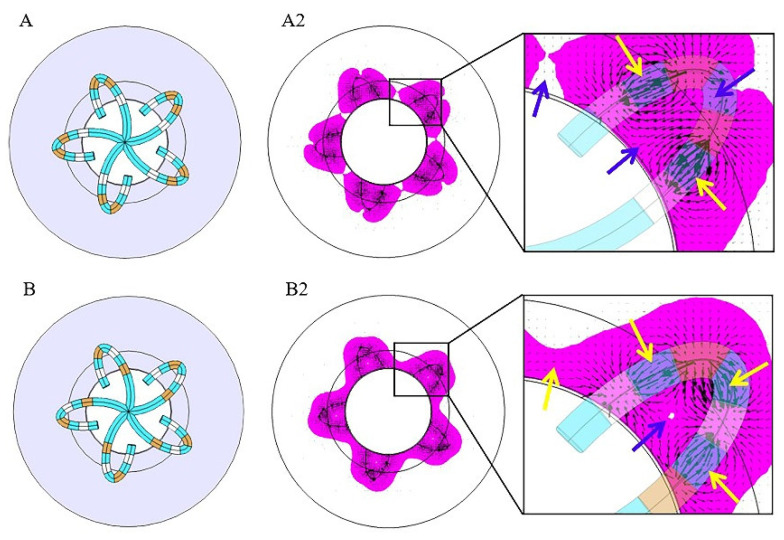
Horizontal distribution of the electric field vector of modes (**A**,**B**). (**A2**,**B2**) are sectional views of the effective lesion area (magenta) on the myocardial surface. The black arrow represents the electric field vector. Blue arrows represent electric field cancellation, and yellow arrows represent electric field superposition. Magenta represents the effective lesion area.

According to Figure 4 and Figure 5 and Table 2 and Table 3, the discharge mode of PFA directly affects the electric field distribution in the myocardium. To explore the mechanism, we further analyzed the electric field distribution of the discharge modes and added the electric field vector (as shown in Figure 7) on the basis of Figure 4A2,B2. The black arrow represents the electric field vector. A larger arrow indicates a higher electric field intensity.

Figure 7 shows that the electric field vector starts from the positive electrodes and ends at the negative electrodes. However, due to the simultaneous discharge of multiple electrodes, electric fields will interfere with each other, thus resulting in the cancellation or superposition of electric fields in some specific areas. Mode A cannot form a continuous ELA between adjacent splines because those areas are just in the middle of two homopolar electrodes, and the electric field is cancelled, thus causing the electric field intensity to significantly decrease out of the scope of ELA (as shown in Figure 7A2). The lesion gap in mode B is caused by electric field cancellation in the middle of two electrodes with the same polarity (as shown in Figure 7B2). In addition to electric field cancellation, electric field superposition will also occur. Mode B can form a continuous circumferential lesion because electrodes of different polarities are located between splines (as shown in Figure 7B2).

**Figure 8:**
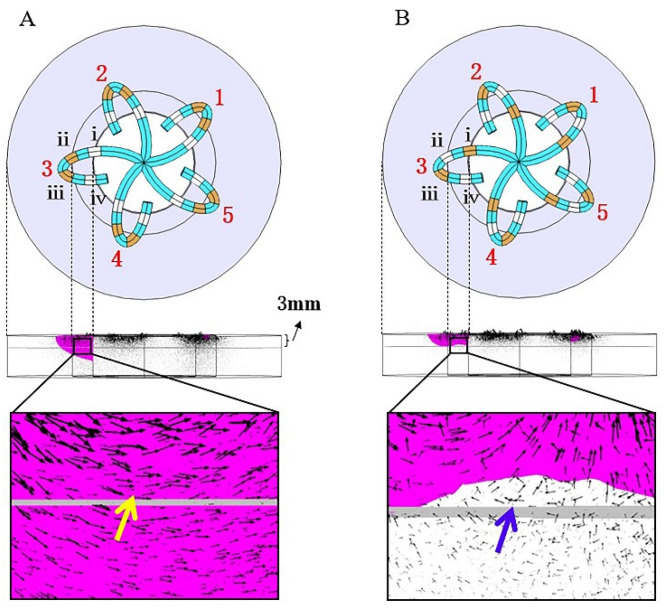
The spatial distribution of the electric field vector of the modes (**A**,**B**). The black arrow represents the electric field vector. Blue arrows represent electric field cancellation, and yellow arrows represent electric field superposition. Magenta represents the effective lesion area.

Figure 8 shows the spatial distribution of the electric field vectors for modes A and B. Due to the arrangement of the electrodes, the electric field vectors on each spline of mode A are emitted by electrodes ii and iii, which point to electrodes i and iv. Their directions are essentially the same, pointing from the outer myocardium to the inner myocardium, thus forming electric field superposition (as shown in Figure 8A), which slows down the decreasing speed of the electric field intensity of mode A. Therefore, Mode A can have a large lesion depth (6.1 mm). For the same reason, the electric field vectors of mode B are emitted by electrodes i and iii, which point to electrodes ii and iv. Due to the fact that their directions are essentially opposite, and there is a large area of electric field cancellation (as shown in Figure 8B), the lesion depth of mode B is small (3.2 mm).

According to the above analysis, we know that the electric field distribution follows a rule: “ electric field cancellation will occur between two electrodes with the same polarity, and electric field superposition will occur between two electrodes with opposite polarities”. The electric field cancellation or superposition area can be changed by adjusting the electrode arrangement and discharge mode to obtain the desired electric field distribution.

**Figure 9:**
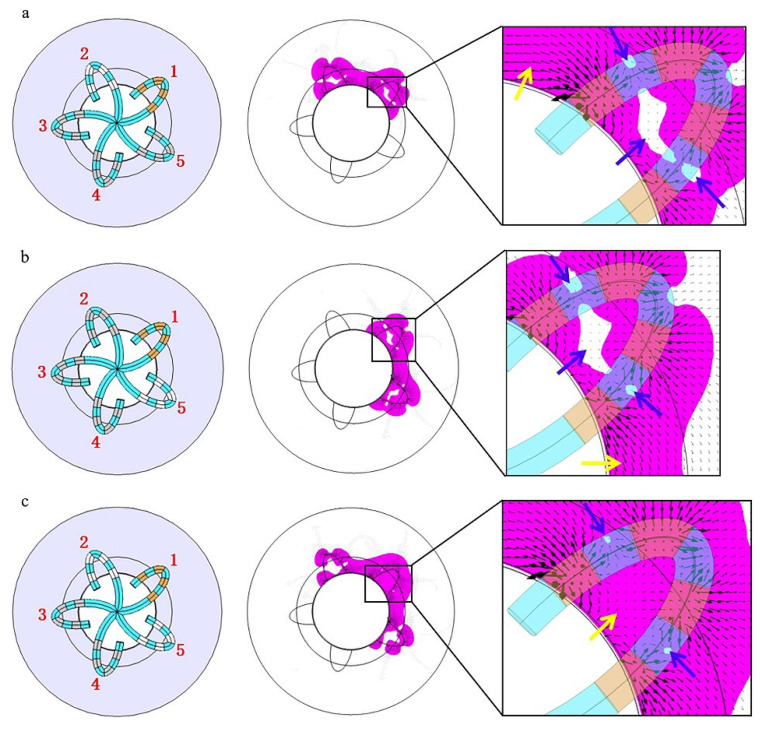
The evolution process of electrode arrangement and effective lesion area of mode C. (**a**) Spline 1 is connected to the positive terminal and spline 2 is grounded; (**b**) spline 1 is connected to the positive terminal, and spline 5 is grounded; (**c**) spline 1 is connected to the positive terminal, and splines 2 and 5 are grounded at the same time. The black arrow is the electric field vector. Blue arrows represent electric field cancellation and yellow arrows represent electric field superposition. Magenta represents the effective lesion area.

Take the problem of using flower catheters to form a uniform, continuous, and transmural circumferential lesion at the pulmonary vein orifice as an example. It is known that mode A can cause a deeper lesion, but the lesion is not continuous between adjacent splines. If we connect the four electrodes on spline 1 to the positive terminal and set the four electrodes on splines 2 or 5 as the ground, the area between the splines will be in a state of electric field superposition (yellow arrows in Figure 9a,b). Additionally, due to the same polarity of the four electrodes on spline 1, severe electric field cancellation also occurs internally (blue arrow in Figure 9a,b). However, the electric field directions (black arrow) in Figure 9a,b are basically the same. If the two electric fields are combined, the superposition of the electric fields inside spline 1 can be realized, and the final result is shown in Figure 9c. If the positive terminal is successively added to the five splines, and the two adjacent splines are grounded each time, this discharge mode is mode C. Therefore, using the rule of electrode arrangement and electric field distribution, we can optimize the discharge mode to achieve precise ablation.

### 4.2. Inspirations for the Clinical Application of PFA

A large number of PFA clinical trials using flower catheters have been carried out and achieved good results [17,21,29]. We only found the pulse amplitude (1800–2000 V biphasic pulses) and ablation strategies (catheter rotation) used in the trials from these literatures. Unfortunately, there is no specific description of the discharge mode of the flower catheter in these literatures, and we can only infer one mode (mode D of this manuscript) from [30].

To explore the lesion differences caused by the different discharge modes of the flower catheter, we proposed three specific discharge modes (A, B, and C) and compared them with mode D from [30]. The results show that the lesions generated by different discharge modes can meet different requirements. Without rotating the catheter, modes B, C, and D can form a continuous circumferential lesion with depths of 1.6 mm, 2.7 mm, and 0.7 mm, respectively, which are suitable for shallow targets ablation. Although the lesion depth of mode B is shallower than that of mode C, the ablation rate in mode B is faster and can be completed in one discharge, whereas mode C requires five discharges. When combined with two rotations, modes A, C, and D can form a continuous and transmural lesion (the depths are 5.2 mm, 4.7 mm, and 4.0 mm, respectively) which can achieve circumferential pulmonary vein isolation.

Overall, the results of this study can help improve PFA procedures and have significant reference for the clinical application of multielectrode catheters in PFA.

### 4.3. Limitations

This study also had some limitations. First, myocardial fibers are anisotropic, but to simplify the problem, we considered the myocardial tissue to be isotropic; thus, the conductivity is the same in all directions.

Second, the purpose of this study was to clarify the effect of the discharge mode of the multielectrode catheter on the pulse electric field distribution, so the model constructed in this manuscript was static (tissue conductivity remained constant) and could not reflect the influence of multiple pulses on electroporation.

Third, we only considered the electric field distribution when the catheter was horizontally attached to the myocardium, which was the ideal situation. In practice, the catheter may tilt, creating a gap between the catheter and the myocardium, which may affect the PFA results.

In the follow-up study, we will further improve these aspects and verify the simulation results through animal experiments.

## 5. Conclusions

The simulation results show that, for multielectrode catheters, the discharge mode directly affects the electric field distribution in the myocardium. Taking the flower catheter as an example, four discharge modes (A, B, C, and D) were simulated (C and D require sequential discharge) in this manuscript, among which modes B, C, and D could form a continuous circumferential ablation lesion without rotating the electrode catheter, with widths of 10.6 mm, 11.8 mm, and 11.5 mm, respectively, and depths of 1.6 mm, 2.7 mm, and 0.7 mm, respectively. Although mode B had the shallowest lesion, it could be completed in one discharge with the fastest ablation speed. Under the condition of rotating the catheter twice, the four discharge modes (A, B, C, and D) could form a continuous ablation lesion with widths of 10.8 mm, 10.6 mm, 11.8 mm, and 11.5 mm, respectively, and depths of 5.2 mm, 2.7 mm, 4.7 mm, and 4.0 mm, respectively. During the implementation of PFA, an appropriate discharge mode can be selected according to the requirements of the ablation depth and width. This manuscript also deeply analyzed the mechanism of the discharge mode that affects the electric field distribution of the myocardium, which provides enlightenment for the design of the discharge mode of PFA with a multielectrode catheter.

## Figures and Tables

**Table 1:** Material properties related to PFA, in which the myocardial conductivity is 5 kHz and the temperature is 37 °C [31].

Material	Myocardium	Blood	Electrode Pt	Catheter
Conductivity σ (s/m)	0.137	0.70	$4.6\times 10^{6}$	$10^{-5}$
Relative permittivity	$1.28\times 10^{5}$	5250	1	1

**Table 2:** The dimensions of the effective lesion area of the four discharge modes without catheter rotation.

Discharge Modes	Maximum Lesion Width on the Myocardial Surface (mm)	Maximum Continuous Circumferential Lesion Depth (mm)	Maximum Lesion Depth (mm)
A	10.8	0	6.1
B	10.6	1.6	3.2
C	11.8	2.7	5.1
D	11.5	0.7	4.3

**Table 3:** The dimensions of the effective lesion area of the four discharge modes after catheter rotations.

Discharge Mode	Maximum Continuous Circumferential Lesion Depth (mm)
A	5.2
B	2.7
C	4.7
D	4.0

## Data Availability

The datasets used and/or analyzed during the current study are available from the corresponding author on reasonable request.
